# The V-shaped curve relationship between fasting plasma glucose and human serum albumin in a large health checkup population in China

**DOI:** 10.1186/s12902-023-01441-z

**Published:** 2023-09-11

**Authors:** Chenxu Wang, Lei Cao, Wendan Mei, Yicheng Fang, Xia Ren, Jian Hu, Fan Su, Grace Tavengana, Mingfei Jiang, Huan Wu, Yufeng Wen

**Affiliations:** 1 School of Public Health, Wannan Medical College, 22 West Wenchang Road, Wuhu, 241002 Anhui Province People’s Republic of China; 2 School of Clinical Medicine, Wannan Medical College, 22 West Wenchang Road, Wuhu, 241002 Anhui Province People’s Republic of China; 3 School of Laboratory Medicine, Wannan Medical College, 22 West Wenchang Road, Wuhu, 241002 Anhui Province People’s Republic of China

**Keywords:** Fasting plasma glucose, Human serum albumin, Genders, Ages, V-shaped curve

## Abstract

**Background:**

This study aimed to investigate the relationship between fasting plasma glucose (FPG) and human serum albumin (HSA) in a large health checkup population in China.

**Methods:**

In this cross-sectional health checkup study, we enrolled a population of 284,635 subjects from Wuhu between 2011 and 2016. All participants completed the physical examination, blood biochemical examination, and blood routine examination.

**Results:**

The prevalence of diabetes in men and women was 6.11% and 2.98%, respectively. The average level of HSA and FPG was significantly higher in men than in women (48.44 ± 3.25 vs. 47.14 ± 3.22, P < 0.0001; 5.50 ± 1.26 vs. 5.26 ± 0.94, P < 0.0001). There were significant differences in blood biochemistry and blood routine values by gender. After adjusting for confounding factors, the results showed that FPG and HSA were a V-shaped curve, and the threshold value of HSA was 40.7 mmol/L. FPG and HSA still showed a V-shaped curve after stratification by gender and age. In the male group, FPG decreased with HSA when HSA<42.3 mmol/L, and increased when HSA ≥ 42.3 mmol/L. In the female group, FPG decreased with HSA when HSA<35.7 mmol/L, and increased when HSA ≥ 35.7 mmol/L. In the age<65 group, FPG decreased with HSA when HSA<37.5 mmol/L, and increased when HSA ≥ 37.5 mmol/L. In the age ≥ 65 group, FPG decreased with HSA when HSA<43.2 mmol/L, and increased when HSA ≥ 43.2 mmol/L.

**Conclusions:**

A V-shape relationship exists between fasting plasma glucose and human serum albumin among the Chinese health checkup population studied.

**Supplementary Information:**

The online version contains supplementary material available at 10.1186/s12902-023-01441-z.

## Background

Albumin is a protein synthesized in the liver and constitutes more than half of the serum proteins. Albumin has important antioxidant activity, anti-inflammatory and anti-coagulant effects [1], and it is also a useful marker for assessing nutritional status [2]. Some previous studies have shown a positive association between higher dietary protein intake and HSA levels [3–5], while other newer studies do not validate that such a relationship exists. Studies have shown that it rarely changes with dietary patterns, except in the case of reduced protein consumption accompanied by adequate caloric intake [6]. A high-protein diet has been associated in some studies with an increased incidence of diabetes [7, 8]. A possible reason for such a relationship might be due to the fact that high protein intake is accompanied by stimulation of glucagon and insulin, high glycogen turnover, and increased gluconeogenesis [9]. It is reasonable to assume that there may be certain relationship between diabetes and HSA concentration. Fasting plasma glucose (FPG) concentration is the most direct indicator of diabetes, and there may be certain association between FPG and HSA concentration.

Since it is unclear whether there is some relationship between FPG and HSA concentration, studying their relationship would be helpful for early detection, diagnosis, and treatment of diabetes. Therefore, it remains crucial to further clarify their relationship. In summary, in this investigation, we conducted a cross-sectional study in a large health screening population in China to understand the relationship between FPG and HSA concentration.

## Methods

### Study participants

In this cross-sectional study, we enrolled a population of 432,430 subjects who had the physical examination at the Center of Health Examination, Yijishan Hospital in Wuhu city from 2011 to 2016. The inclusion criteria were: (1) subjects within the age range of 18–98 years; (2) available data on gender, age, height, weight, systolic blood pressure (SBP), diastolic blood pressure (DBP), total cholesterol (TC), triglycerides (TG), high-density lipoprotein cholesterol (HDL-C), low-density lipoprotein cholesterol (LDL-C), hemoglobin (HGB), white blood cells (WBC), red blood cells (RBC), human serum albumin (HSA), and fasting plasma glucose (FPG); and (3) without a history of anti-diabetes treatment. The exclusion criteria were: (1) subjects with incomplete clinical data, and missing data; (2) subjects with a history of diabetes; and (3) subjects with morbidity history of kidney disease, gout, and cancer. All subjects underwent the physical examination, blood biochemistry, and blood tests. Diabetes mellitus was diagnosed based on FPG of ≥ 126 mg/dL (7.0 mmol/L) (fasting time 8-12 h) [10], or if the subject was taking medication to control blood glucose at the time. The study was consistent with Helsinki guidelines of the Helsinki Declaration of World Medical Association. This study was approved by the Ethics Committee of Wannan Medical College.

### Questionnaire recorded

The baseline information of the study subjects included general information such as gender, age, occupation, and education. Medical history information included hypertension, diabetes mellitus, dyslipidemia or kidney disease, gout, and cancer. Whether taking antihypertensive drugs, glucose-lowering drugs, lipid-lowering drugs, etc. In addition, information on lifestyle habits such as history and frequency of smoking, history and frequency of alcohol consumption, dietary habits, and weekly exercise time were also collected and compiled [11].

### Physical examination

Under the guidance of the WHO and the International Society of Hypertension [12], trained specialists used the usual methods of measuring height, weight, and blood pressure. For height measurement, the subject should stand barefoot on the floor of the height measuring machine with the body naturally extended, head straight, eyes straight ahead, upper arms naturally down, and legs straight. When measuring weight, a barefoot person should naturally stand in the middle of the weight pedal and wait for the scale number to stabilize. BMI was measured by dividing the square of height (m) by weight (kg). A mercury column sphygmomanometer was used to measure SBP and DBP. subjects were recommended to sit still for 5 min before taking their blood pressure [13].

### Laboratory measurements

Fasting venous blood was obtained in the morning. Biochemical parameters (TC, TG, HDL-C, LDL-C, and FPG) were measured using a Hitachi 7600 automatic biochemistry analyzer. Routine blood tests for HGB, WBC, and RBC were performed using a Beckman Coulter Automated CBC analyzer (Beckman Coulter, Inc, Fullerton, CA, USA).

### Statistical analysis

Data analyses were done using SPSS 20.0 software and R-Project 3.6.3. Mean ± standard deviation was used to express continuous variables, while frequencies and ratios were used to express discrete variables. Males and females were t-tested for demographics, physical examination, biochemistry, and blood count. The prevalence of diabetes mellitus was compared across gender using chi-square test. The relationship between FPG and HSA was validated using the generalized smooth spline method, and the node positions were automatically generated in the generalized additive model using the R software MGCV. The correlation between FPG and HSA was further investigated by linear regression of HSA nodal sites for each segment of HSA, adjusted for gender, age, BMI, SBP, DBP, TC, HGB, WBC, and RBC. Then the correlation between FPG and HSA was investigated using the generalized smoothing spline method and adjusted accordingly and linear regression was performed on the correlation with HSA. All p values were two-tailed, with a significance level of 0.05.

## Results

### Characteristics of subjects

Table 1 showed that a total of 284,635 subjects were included in this study that comprised 165,496 men (58.14%) and 119,139 women (41.86%), with the mean age of 48.19 ± 13.76 and 46.97 ± 13.51 years, respectively. The prevalence of diabetes in men and women was 6.11% and 2.98%, respectively. The average level of HSA and FPG was significantly higher in men than in women (48.44 ± 3.25 vs. 47.14 ± 3.22, P < 0.0001; 5.50 ± 1.26 vs. 5.26 ± 0.94, P < 0.0001). There were significant differences in blood biochemistry and blood routine values by gender.

**Table 1:** Comparison of demographic characteristics and biochemical indicators by genders

Variable	Male(*n =* 165,496)	Female(*n* = 119,139)	*t/χ* ^*2*^	*P*
AGE	48.19 ± 13.76	46.97 ± 13.51	-23.43	< 0.0001
BMI	24.52 ± 3.11	22.78 ± 3.12	-146.90	< 0.0001
SBP	122.09 ± 16.19	115.38 ± 17.03	-106.64	< 0.0001
DBP	79.67 ± 9.82	74.21 ± 9.24	-149.80	< 0.0001
TC	4.65 ± 0.90	4.60 ± 0.90	-15.91	< 0.0001
TG	1.78 ± 1.39	1.25 ± 0.88	-115.57	< 0.0001
HDL-C	1.28 ± 0.32	1.51 ± 0.35	179.86	< 0.0001
LDL-C	2.59 ± 0.78	2.53 ± 0.77	-20.69	< 0.0001
HGB	150.20 ± 11.18	127.70 ± 11.36	-526.19	< 0.0001
WBC	6.59 ± 1.65	6.01 ± 1.54	-94.69	< 0.0001
RBC	4.90 ± 0.43	4.31 ± 0.36	-389.14	< 0.0001
HSA	48.44 ± 3.25	47.14 ± 3.22	-105.27	< 0.0001
FPG	5.50 ± 1.26	5.26 ± 0.94	-57.19	< 0.0001
Diabetes	10,105(6.11%)	3549(2.98%)	1483.17	< 0.0001

BMI, body mass index; SBP, systolic blood pressure; DBP, diastolic blood pressure; TC, total cholesterol; TG, triglycerides; HDL-C, high-density lipoprotein cholesterol; LDL-C, low-density lipoprotein cholesterol; HGB, hemoglobin; WBC, white blood cells; RBC, red blood cells; HSA, human serum albumin; FPG, fasting plasma glucose

### Relationship between FPG and HSA

**Fig. 1:**
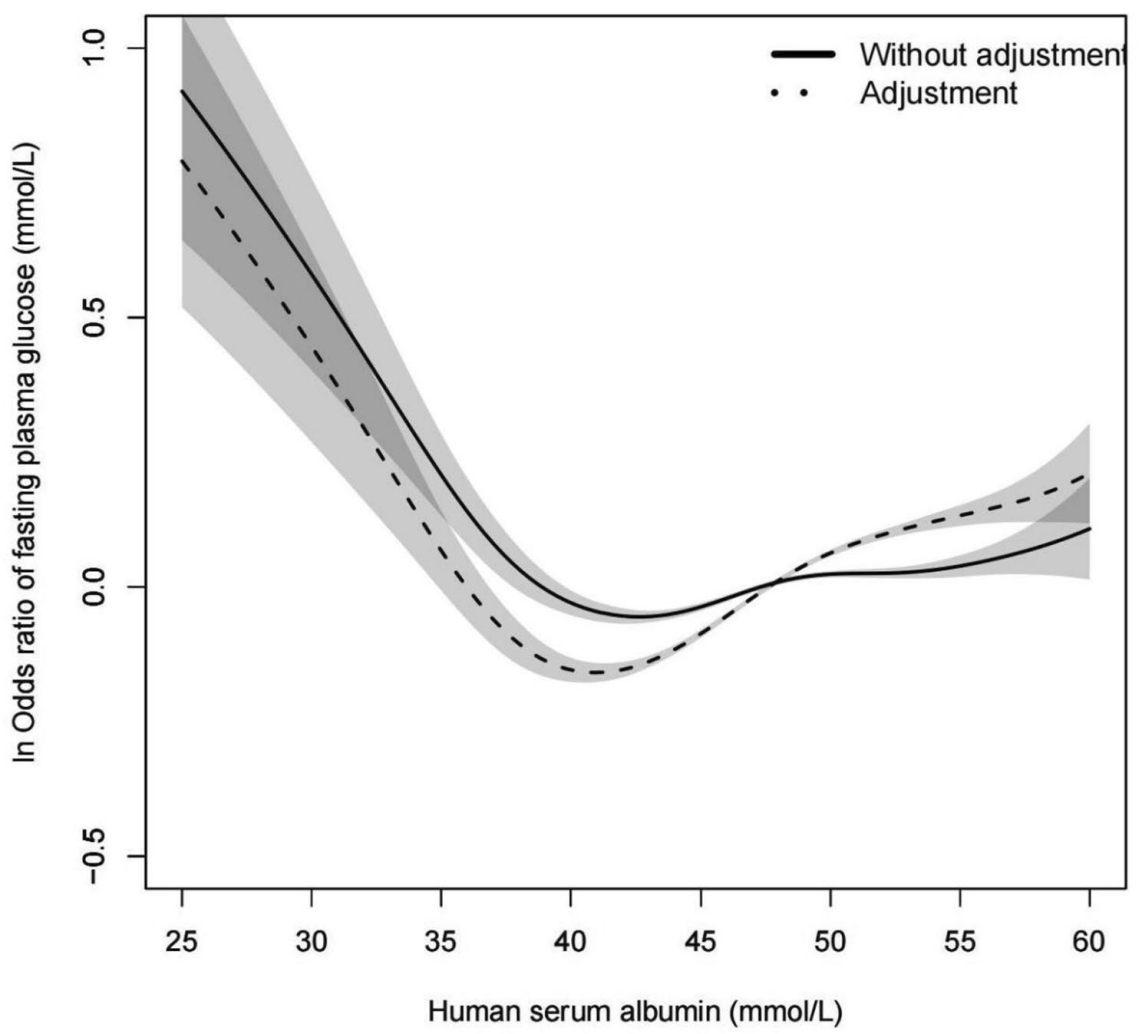
Relationship between FPG and HSA. (The horizontal axis represents various levels of HSA and the vertical axis is ln odds ratio of FPG). Solid line: without adjustment; dotted line: adjustment for gender, age, BMI, SBP, DBP, TC, HGB, WBC, and RBC. Shaded area shows the 95% confidence interval

Figure 1 showed a V-shaped curve relationship between FPG and HSA, the threshold value of HSA was 40.7 mmol/L. FPG decreased with HSA when HSA<40.7 mmol/L, and increased when HSA ≥ 40.7 mmol/L, after adjustment for gender, age, BMI, SBP, DBP, TC, HGB, WBC, and RBC. These results are presented on Table 2.

**Table 2:** Association between FPG and HSA by linear regression model

HSA	Unadjusted				Adjusted^a^			
(mmol/L)	B	S.E	*t*	*P*	B	S.E	*t*	*P*
HSA<40.7	-0.066	0.011	-6.030	< 0.0001	-0.058	0.011	-5.410	< 0.0001
HSA ≥ 40.7	0.008	0.001	11.900	< 0.0001	0.024	0.001	34.640	< 0.0001

^a^ Adjusted for gender, age, BMI, SBP, DBP, TC, HGB, WBC, and RBC

### Relationship between FPG and HSA in different genders

**Fig. 2:**
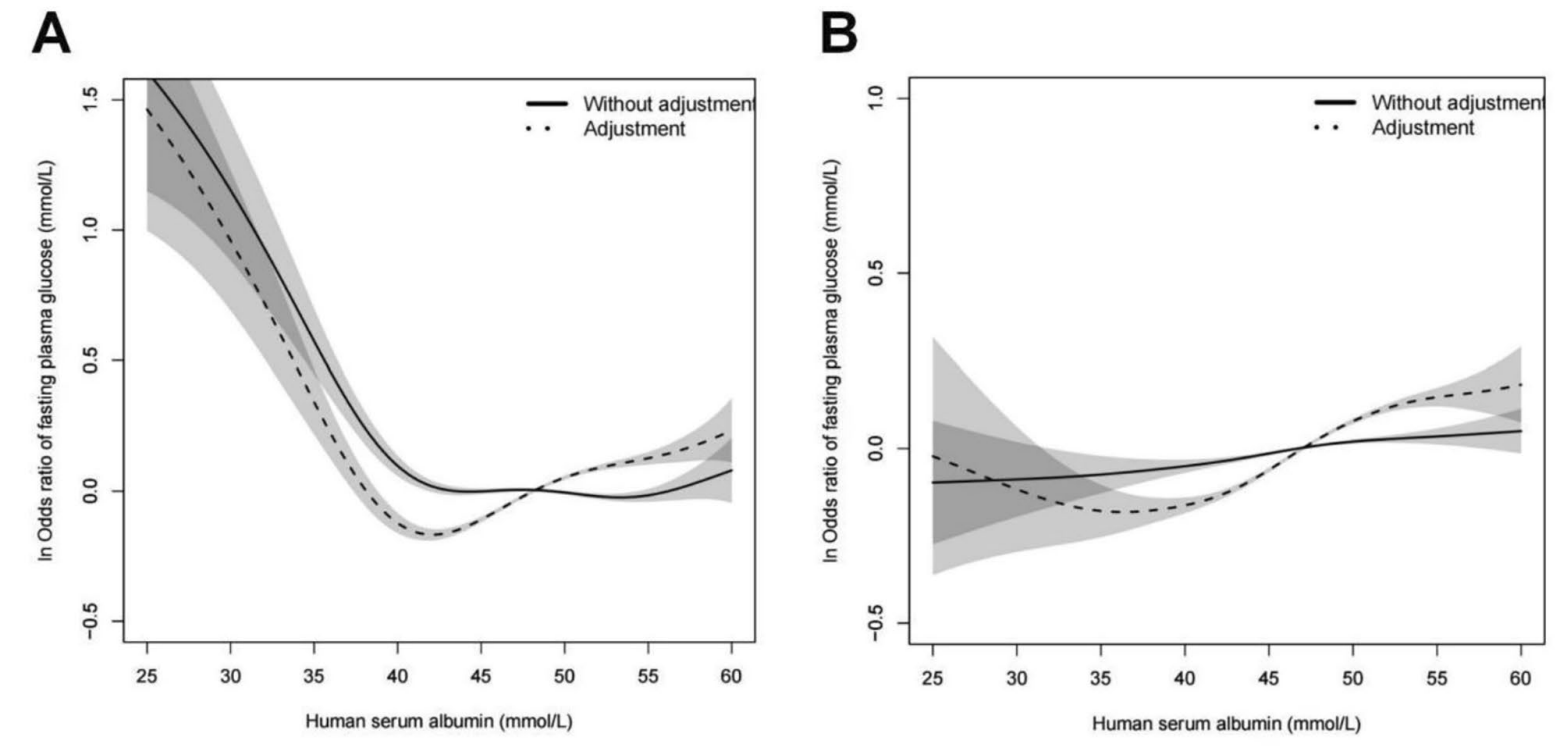
Relationship between FPG and HSA in men (**A**) and women (**B**) (The horizontal axis represents various levels of HSA and the vertical axis is ln odds ratio of FPG). Solid line: without adjustment; dotted line: adjustment for age, BMI, SBP, DBP, TC, HGB, WBC, and RBC. Shaded area shows the 95% confidence interval

Figure 2 showed that through gender stratification and after adjustment for age, BMI, SBP, DBP, TC, HGB, WBC, and RBC, a V-shaped curve relationship was established between FPG and HSA in the male and female groups, respectively. In the male group, FPG decreased with HSA when HSA<42.3 mmol/L, and increased when HSA ≥ 42.3 mmol/L; In the female group, FPG decreased with HSA when HSA<35.7 mmol/L, and increased when HSA ≥ 35.7 mmol/L. These data are shown on Table 3.

**Table 3:** Association between FPG and HSA by linear regression model in different genders

Gender	HSA	Unadjusted				Adjusted^a^			
	(mmol/L)	B	S.E	*t*	*P*	B	S.E	*t*	*P*
Male	HSA<42.3	-0.070	0.011	-6.120	< 0.0001	-0.064	0.011	-5.630	< 0.0001
	HSA ≥ 42.3	-0.002	0.001	-1.840	0.0664	0.026	0.001	24.460	< 0.0001
Female	HSA<35.7	-0.107	0.049	-2.180	0.0313	-0.114	0.049	-2.320	0.0219
	HSA ≥ 35.7	0.006	0.001	7.320	< 0.0001	0.024	0.001	29.090	< 0.0001

^a^ Adjusted for age, BMI, SBP, DBP, TC, HGB, WBC, and RBC

### Relationship between FPG and HSA in different ages

**Fig. 3:**
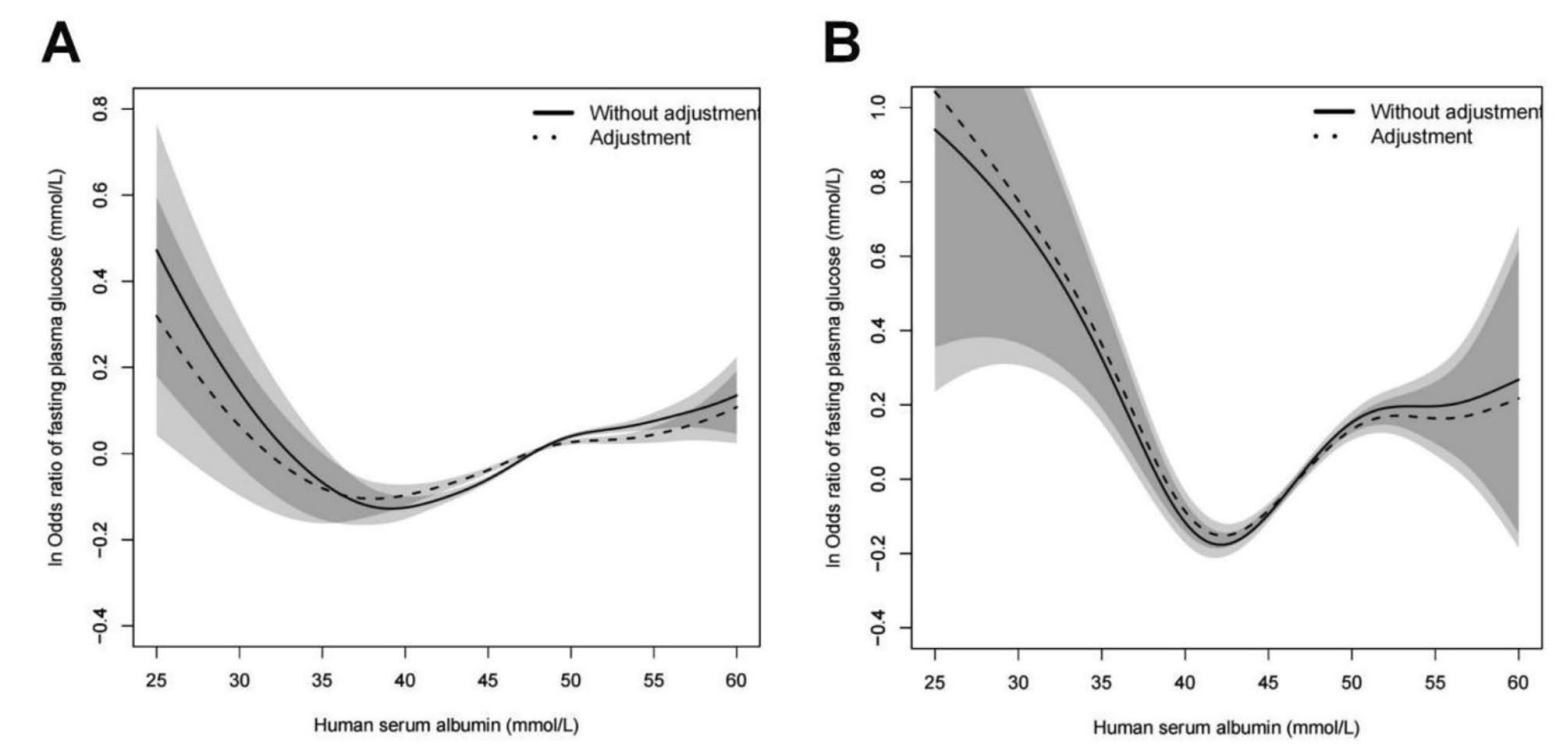
Relationship between FPG and HSA in age<65 (**A**) and age ≥ 65 (**B**). (The horizontal axis represents various levels of HSA and the vertical axis is ln odds ratio of FPG). Solid line: without adjustment; dotted line: adjustment for gender, BMI, SBP, DBP, TC, HGB, WBC, and RBC. Shaded area shows the 95% confidence interval

Figure 3 showed that through age stratification and after adjustment for gender, BMI, SBP, DBP, TC, HGB, WBC, and RBC, a V-shaped curve relationship was established between FPG and HSA in the age<65 and age ≥ 65 groups, respectively. In the age<65 group, FPG decreased with HSA when HSA<37.5 mmol/L, and increased when HSA ≥ 37.5 mmol/L; In the age ≥ 65 group, FPG decreased with HSA when HSA<43.2 mmol/L, and increased when HSA ≥ 43.2 mmol/L. These data are shown on Table 4.

**Table 4:** Association between FPG and HSA by linear regression model in different ages

Age	HSA	Unadjusted				Adjusted^a^			
	(mmol/L)	B	S.E	*t*	*P*	B	S.E	*t*	*P*
age<65	HSA<37.5	-0.114	0.027	-4.170	< 0.0001	-0.108	0.027	-3.950	< 0.0001
	HSA ≥ 37.5	0.015	0.001	23.070	< 0.0001	0.010	0.001	14.830	< 0.0001
age ≥ 65	HSA<43.2	-0.059	0.012	-5.030	< 0.0001	-0.057	0.012	-4.870	< 0.0001
	HSA ≥ 43.2	0.041	0.003	12.520	< 0.0001	0.034	0.003	10.510	< 0.0001

^a^ Adjusted for gender, BMI, SBP, DBP, TC, HGB, WBC, and RBC

## Discussion

Given the cross-sectional nature of our study, the possible mechanisms behind our findings may only be speculated. This study found a V-shaped curve relationship between FPG and HSA, in which FPG concentrations first decreased and then increased with increasing HSA concentrations. After stratification by gender and age [14, 15], a V-shaped curve relationship between FPG and HSA was still established. The cut-off value of HSA in the male and female groups was 42.3 mmol/L and 35.7 mmol/L, respectively. And in the age<65 and age ≥ 65 groups was 37.5 mmol/L and 43.2 mmol/L, respectively.

Previous studies have shown that serum albumin is the most abundant circulating protein and its antioxidant properties are significant [16, 17]. The protective effect of anterior serum albumin concentration on impaired glycemic control may be due to its antioxidant effect, which binds to reactive oxygen species (ROS), thus preventing ROS from disrupting insulin signaling pathways and failing to induce cytotoxicity in pancreatic β-cells, thereby reducing the development of diabetes [18]. Another possible factor could be the hydration status of the participants, which is considered to be associated with higher glucose values, while dehydration is currently recognized as the only cause of high HSA [19], so it can be assumed that HSA values are higher and FPG is lower in the dehydrated state. This is consistent with the results of the first half of the V-shaped curve found in this study, and explains well the possible reasons for the decrease in FPG with increasing HSA concentrations in the early stages. The dangerous role of posterior serum albumin concentration on glycemic control may be due to the fact that higher serum albumin tends to reflect overnutrition [20], and the most direct outcome of overnutrition is obesity, which is an independent risk factor for the development of the metabolic syndrome and diabetes [21, 22]; therefore, higher serum albumin concentration may cause elevated blood glucose concentration. This is consistent with the results of the second half of the V-shaped curve found in this study, and explains well the possible reasons for the increase in FPG in the later stage as HSA concentration increases.

In summary, the first half of the V-shaped curve found in this study may be due to the fact that as the concentration of HSA gradually increases, its antioxidant capacity also becomes stronger, thus preventing ROS from disrupting the insulin signaling pathway, reducing the development of diabetes and leading to lower FPG concentration. It is also possible that this is due to the hydration status of the participants. The second half of the V-shaped curve may be due to the fact that after a certain threshold is exceeded, higher concentration of HSA tends to reflect overnutrition. Thus the incidence of metabolic syndrome and diabetes is increased, leading to higher FPG concentration.

## Limitations of the study

There are several limitations to the study. First, it is a cross-sectional survey and does not address causality. However, this study used a large sample of clinical epidemiological surveys, and its findings have a high degree of objectivity. Second, we compared the baseline characteristics of the included and excluded participants in the supplementary material and found differences in the two samples. This may be due to the fact that large sample sizes improve the precision and efficacy of statistical tests, making it easier to detect differences between the two samples. Third, the study was based on information obtained from the same hospital, which would limit its representativeness. Fourth, the subjects of this thesis are health checkups, whose characteristics differ from those of community residents, which makes the results of this study representative. In the future, we will further broaden the research area and develop multidisciplinary cooperation. More studies are needed to confirm our findings and to identify the possible mechanisms involved.

## Conclusions

The relationship between FPG and HSA presents a V-shaped curve among the Chinese health checkup population studied. After stratification by gender and age, in the male and female groups, in the age<65 and age ≥ 65 groups still present a V-shaped curve irrespective of adjustments for BMI, SBP, DBP, TC, HGB, WBC, and RBC.

### Electronic supplementary material

Below is the link to the electronic supplementary material.

Supplementary Material 1

## Data Availability

The data of participants are collected by the authors and uploaded to the database, which makes it easier for the authors to use the data in the process of analyzing data and writing manuscripts. This kind of database system can conveniently shield the data irrelevant to the experiment and effectively protect the privacy of participants. The data that support the findings of this study are available from the Health Management Center at the First Affiliated Hospital of Wannan Medical College in Wuhu, China but restrictions apply to the availability of these data, which were used under license for the current study, and so are not publicly available. Data are however available from the authors upon reasonable request and with permission of Health Management Center at the First Affiliated Hospital of Wannan Medical College in Wuhu, China. If someone wants to request the data from this study, they can contact Yufeng Wen (corresponding author).

## Abbreviations

FPG: Fasting plasma glucose
HSA: Human serum albumin
BMI: Body mass index
SBP: Systolic blood pressure
DBP: Diastolic blood pressure
TC: Total cholesterol
TG: Triglycerides
HDL-C: High-density lipoprotein cholesterol
LDL-C: Low-density lipoprotein cholesterol
HGB: Hemoglobin
WBC: White blood cells
RBC: Red blood cells
WHO: World Health Organization
