# Supplementary material for: The V-shaped curve relationship between fasting plasma glucose and human serum albumin in a large health checkup population in China
Source: BMC Endocr Disord. 2023 Sep 11;23:192. doi: 10.1186/s12902-023-01441-z (PMC10494399; doi:10.1186/s12902-023-01441-z)
Supplement: Supplementary file 1 — Supplementary Material 1 [file 12902_2023_1441_MOESM1_ESM.docx]

| Variable | Included(***n=***284635) | Excluded**(*n*=**147795) | ***t/χ^2^*** | ***P*** |
| --- | --- | --- | --- | --- |
| AGE | 47.68±13.67 | 39.54±13.18 | -187.44 | <0.0001 |
| BMI | 23.79±3.23 | 23.11±3.38 | -55.36 | <0.0001 |
| SBP | 119.28±16.87 | 115.66±14.99 | -61.29 | <0.0001 |
| DBP | 77.38±9.95 | 76.02±9.40 | -38.68 | <0.0001 |
| TC | 4.63±0.90 | 4.66±0.92 | 7.22 | <0.0001 |
| TG | 1.56±1.23 | 1.66±1.50 | 15.34 | <0.0001 |
| HDL-C | 1.37±0.35 | 1.36±0.35 | -6.54 | <0.0001 |
| LDL-C | 2.56±0.78 | 2.58±0.79 | 5.34 | <0.0001 |
| HGB | 140.78±15.81 | 143.71±15.67 | 53.10 | <0.0001 |
| WBC | 6.35±1.63 | 6.46±1.67 | 19.17 | <0.0001 |
| RBC | 4.65±0.50 | 4.76±0.50 | 58.25 | <0.0001 |
| HSA | 47.89±3.30 | 48.51±3.25 | 54.30 | <0.0001 |
| FPG | 5.40±1.14 | 5.27±1.01 | -31.07 | <0.0001 |
| Diabetes | 13654(4.80%) | 3382(2.29%) | 1617.85 | <0.0001 |

**Table S1** Comparison of baseline characteristics of included and excluded participants

BMI, body mass index; SBP, systolic blood pressure; DBP, diastolic blood pressure; TC, total cholesterol; TG, triglycerides; HDL-C, high-density lipoprotein cholesterol; LDL-C, low-density lipoprotein cholesterol; HGB, hemoglobin; WBC, white blood cells; RBC, red blood cells; HSA, human serum albumin; FPG, fasting plasma glucose.
